# A Handbook on the Diseases of the Nervous System

**Published:** 1886-11

**Authors:** 


A Handbook on the Diseases of the Nervous System. By James Ross, M. D., F. R. C. P., LL. D., Senior Assistant Physician to the Manchester Royal Infirmary, etc. Octavo, 726 pages, 184 illustrations. Cloth, $4.50; leather, $5.50. Philadelphia: Lea Brothers & Co. 1885.

This work of foreign authorship is an appropriate addition to the labors of American writers upon the diseases of the nervous system in the recently issued fifth and closing volume of Pepper's System of Medicine.

It may be considered as a general summing up of theoretic and practical views recognized in the progressive neurology of the present day, based upon a clinical classification.

While the first three chapters present the result of investigations bearing upon histology and physiology from the standpoint of the specialist, which may not appear as essential for the comprehension of the practical details in which the members of the medical profession generally are most interested, yet this constitutes the basis upon which rest all the departures from the normal performance of the functions of the nervous system.

In the seventh chapter, the author grasps the whole correlation of organs by the concise statement that the movements of the internal organs are regulated chiefly by the ganglia and plexuses of the sympathetic system, but these are so inextricably connected with the cerebro-spinal system that it is impossible to draw any line of demarcation between the two nervous mechanisms.

The sympathetic system of man consists of a vertebral and prevertebral portion. The cerebro-spinal nerves communicate with the cord of the sympathetic at their exit from the cranium and vertebral canal. The sympathetic branches of distribution accompany the arteries, so that all the organs of the body are supplied by sympathetic nerves.

The general treatment of nervous diseases is considered in the eighth chapter, and is divided into that which is directed to the prevention of disease, the removal of the exciting cause and the anatomical cause of disease, with the alleviation of serious symptoms.

Special pathology of the nervous system forms a distinct heading for an elaborate disquisition upon the various nervous affections, running through twenty chapters, and the book closes with a discussion of the general principles of diagnosis of the diseases of the nervous system.

